# Toxicity Minimized Cryoprotectant Addition and Removal Procedures for Adherent Endothelial Cells

**DOI:** 10.1371/journal.pone.0142828

**Published:** 2015-11-25

**Authors:** Allyson Fry Davidson, Cameron Glasscock, Danielle R. McClanahan, James D. Benson, Adam Z. Higgins

**Affiliations:** 1 School of Chemical, Biological and Environmental Engineering, Oregon State University, Corvallis, Oregon, United States of America; 2 Department of Mathematical Sciences, Northern Illinois University, DeKalb, IL, United States of America; The Ohio State University, UNITED STATES

## Abstract

Ice-free cryopreservation, known as vitrification, is an appealing approach for banking of adherent cells and tissues because it prevents dissociation and morphological damage that may result from ice crystal formation. However, current vitrification methods are often limited by the cytotoxicity of the concentrated cryoprotective agent (CPA) solutions that are required to suppress ice formation. Recently, we described a mathematical strategy for identifying minimally toxic CPA equilibration procedures based on the minimization of a toxicity cost function. Here we provide direct experimental support for the feasibility of these methods when applied to adherent endothelial cells. We first developed a concentration- and temperature-dependent toxicity cost function by exposing the cells to a range of glycerol concentrations at 21°C and 37°C, and fitting the resulting viability data to a first order cell death model. This cost function was then numerically minimized in our state constrained optimization routine to determine addition and removal procedures for 17 molal (mol/kg water) glycerol solutions. Using these predicted optimal procedures, we obtained 81% recovery after exposure to vitrification solutions, as well as successful vitrification with the relatively slow cooling and warming rates of 50°C/min and 130°C/min. In comparison, conventional multistep CPA equilibration procedures resulted in much lower cell yields of about 10%. Our results demonstrate the potential for rational design of minimally toxic vitrification procedures and pave the way for extension of our optimization approach to other adherent cell types as well as more complex systems such as tissues and organs.

## Introduction

The conventional cryopreservation approach involves equilibration of cells with relatively low cryoprotective agent (CPA) concentrations (e.g., 10% dimethyl sulfoxide) and slow cooling (~1°C/min) in the presence of extracellular ice prior to storage in liquid nitrogen. This approach is routinely used in many laboratories for cryopreservation of cell cultures after the cells have been brought into suspension. However, cryopreservation of adherent cells may be advantageous for cell types that are difficult to preserve in suspension (e.g., stem cells [1]) or when it is necessary to preserve characteristics of the adherent cultured cells (e.g., neuronal networks [2]). In addition, the ability to cryopreserve cells in the adherent state would enable improvements in experimental workflow by eliminating the need for cell dissociation prior to cryopreservation and replating after thawing. This capability would be particularly useful for slow growing cells such as human embryonic stem cells [3]. The ability to cryopreserve adherent cells would also allow off-the-shelf availability for applications such as drug screening [4] and cell-based biosensors [5]. Conventional slow cooling approaches have been used previously to cryopreserve adherent cells [1, 2, 6–10], but cell recovery post-thaw has typically been low, and it has been suggested that cell-cell and cell-substrate connections make adherent cells particularly susceptible to freezing damage [11–13].

Ice-free cryopreservation, known as vitrification, is a cryopreservation procedure that prevents ice crystal formation in the entire system, not just in the intracellular space, and is a promising method for cryopreservation of adherent cells and tissues [6, 14, 15]. Ice-free cryopreservation requires a balance of extremely high cooling and warming rates and high CPA concentrations. If extremely high cooling and warming rates are achievable, then vitrification is possible even for low CPA concentrations. Conversely, if extremely high CPA concentrations are achievable, then the sample can be vitrified even with low cooling and warming rates. In our case, since adherent cell and tissue samples and their associated culture vessels have a relatively large thermal mass, it is difficult to achieve extremely fast cooling and warming rates. Therefore, successful vitrification of adherent cells and tissues will require high CPA concentrations to prevent ice formation. However, the use of high CPA concentrations increases the likelihood of osmotic damage and CPA-induced cytotoxicity [14, 16, 17].

Osmotic damage arises from the fact that equilibration with and from multimolar CPA concentrations usually is associated with large osmotic gradients driving water fluxes that can cause cell volumes to exceed biophysical limits [18, 19]. Typically, damage of this nature has been avoided using multistep procedures that reduce concentration changes so that osmotic gradients for individual steps are not damaging. Safe protocols can be mathematically determined by coupling knowledge of cellular mass transport kinetics and experimentally determined maximal and minimal volume limits, known in the literature as osmotic tolerance limits [18, 19].

Avoidance of CPA toxicity is considered to be one of the most significant hurdles to successful cryopreservation via vitrification techniques [14, 20]. CPA toxicity is dependent on many factors that include the CPA type (e.g. dimethyl sulfoxide, glycerol, etc…), duration of exposure to CPA, CPA concentration, and the exposure temperature [16, 20–24]. Design of optimal CPA equilibration (addition and removal) procedures requires accounting for all of these interacting factors, which makes rigorous experimental optimization impractical. Thus, several groups have examined the use of mathematical methods to help guide and streamline experimental optimization [25–27].

The simplest CPA toxicity model is that toxicity increases proportionately with exposure time. This model, coupled with the osmotic tolerance limits as state constraints, has been used to optimize protocols utilizing step changes in CPA concentrations [27, 28], as well as protocols where concentrations were allowed to change continuously as a function of time [29, 30]. However, it is reasonable to expect that the damaging action of CPA is concentration dependent. Therefore, we recently proposed a cost function that incorporates both the kinetics and concentration-dependence of toxicity and used published data to determine model parameters [30]. We then numerically determined CPA equilibration procedures that minimize this toxicity cost function for human oocytes, and showed that these procedures are theoretically much less toxic than conventional CPA equilibration procedures [30, 31]. However, these toxicity-minimized procedures have not been tested experimentally.

The purpose of this study was to validate our mathematical optimization approach for designing minimally toxic vitrification procedures. Adherent endothelial cells were chosen as a model system for these studies because endothelial cells are an important component of nearly all tissues, as well as a potential sensing element in cell-based biosensors [5]. To develop a concentration and temperature dependent cost function, kinetic toxicity data was acquired after exposure to glycerol at 21°C and 37°C. This cost function was then used to design procedures for equilibration with a vitrification solution containing 17 molal (mol/kg water) glycerol. The resulting toxicity-minimized procedures resulted in cell yields that were comparable to the control and much better than conventional procedures. Our results demonstrate the potential for model-based design of toxicity-minimized CPA equilibration procedures for vitrification of adherent endothelial cells and lay the groundwork for extension of our optimization approach to other types of adherent cells and tissues.

## Materials and Methods

### Cell Culture

Bovine pulmonary artery endothelial cells were purchased from Cambrex (San Diego, CA). To prepare samples for experiments, cells at passage 6 were thawed and cultured on tissue culture treated plastic T25 flasks in a 5% CO_2_ environment at 37°C, as previously described [32]. After 4 days in culture, the cells were transferred to T75 flasks and cultured for another 3 days. For osmotic tolerance and CPA cytotoxicity experiments, the cells were then seeded into black 96 well plates at a density of 1x10^4^ cells per well and cultured for 3 days before experimentation, at which point they had reached an approximate confluency of 80%. For vitrification experiments, cells were seeded onto 12 mm diameter glass coverslips in a 24 well plate at a density of 2.5x10^4^ cells/well, and cultured for 3 days before experimentation.

### Experimental Solutions

Chemicals were purchased from Mallinkrodt (Hazelwood, MO) unless otherwise noted.

Hypo- and hypertonic solutions for osmotic tolerance experiments were prepared using three different types of stock solutions: isotonic (0.3 Osm/kg) HEPES buffered saline, distilled water and hypertonic (5.3 Osm/kg) stock solutions. Isotonic HEPES buffered saline was made in-house by dissolving salts (0.1 g CaCl_2_, 0.1 g MgCl_2_·H_2_O, 0.2 g KCl, 5.95 g HEPES, and 8 g NaCl) in 1 L of cell culture grade water (Fisher Scientific). Hypertonic stock solutions were made by adding either 855 g sucrose or 87 g NaCl to 500 g of isotonic buffer solution. We assumed that sucrose did not dissociate in solution and that NaCl had a dissociation factor of 1.68 [33] resulting in a final concentration of 5.3 Osm/kg for both stock solutions. All stock solutions were sterilized by filtration. Hypotonic test solutions were prepared by adding sterile distilled water to isotonic buffer to create solution osmolalities of 25, 50, 100, and 200 mOsm/kg. Hypertonic test solutions were prepared by combining 5.3 Osm/kg stock solution with isotonic buffer to create solution osmolalities of 500, 1000, 1500, 2000, 2500, 3000, 3500, and 4000 mOsm/kg. To determine volumes of solutions to combine for each working concentration we used the following equation:

$$M_{f}=\frac{V_{1}\gamma_{w,1}M_{1}+V_{2}\gamma_{w,2}M_{2}}{V_{1}\gamma_{w,1}+V_{2}\gamma_{w,2}},$$ (1)

where *M* is the osmolality (Osm/kg), *V* is the volume of solution to be added to the mixture (L), *γ*_w_ is the mass concentration of water in the solution (kg/L), and the subscripts f, 1 and 2 represent the final mixture and the two stock solutions used to create the final mixture, respectively. Pure water and the isotonic buffer were both assumed to have a water mass concentration of γ_w_ = 1 kg/L. The mass concentration of water in the hypertonic stock solutions was determined using the water mass fraction and the measured density of the solutions, resulting in *γ*_w_ = 0.51 kg/L for the sucrose stock solution and *γ*_w_ = 0.89 kg/L for the NaCl stock solution.

Test solutions for CPA cytotoxicity experiments were prepared by combining a concentrated glycerol stock solution (10 molal) with isotonic HEPES buffered saline. The glycerol stock solution was made by adding 230 g of glycerol and 1.85 g of NaCl to 250 g of isotonic buffer. The additional NaCl was included in this solution to counteract the dilutive effects of glycerol. The volumes of solutions to combine for each working solution concentration were determined using Eq 1, with *γ*_*w*_ = 0.58 kg/L as the mass concentration of water in the glycerol stock solution.

A freezing point depression osmometer (Advanced Micro Osmometer Model 3300, Advanced Instruments, Norwood, MA) was used to confirm the osmolalities of test solutions with nominal concentrations of 1 Osm/kg or less. The measured osmolalities were confirmed to be within 5% of the nominal values reported here.

### Measurement of Cell Viability

The resazurin based metabolic dye PrestoBlue (Invitrogen, Carlsbad, CA) was used to assess cell viability. For experiments in 96 well plates, each well was first washed three times with 90 μL of isotonic buffered saline, being careful not to disturb the adherent cell layer. The final wash volume was combined with 10 μL of PrestoBlue solution and incubated under a foil cover at room temperature for 30 min. At the end of the incubation period, fluorescence was measured on a Victor 3V 1420 plate reader (Perkin Elmer, Watham, MA) using excitation and emission filters of 545 nm and 572 nm, respectively. To determine the background dye fluorescence, 90 μL of buffered saline and 10 μL of PrestoBlue were added to each well of a cell-free plate, and fluorescence was tested after a 30 min incubation. To adjust the procedure for use with 24 well plates, the volumes were simply scaled based on the well surface area.

### Osmotic Tolerance Limits

The osmotic tolerance limits were determined from measurements of the change in cell viability after exposure to a range of anisotonic conditions in a 96 well plate. The initial viability of the cultured endothelial cells was first assessed using the PrestoBlue assay (as described above), followed by removal of the PrestoBlue solution by washing 3 times with isotonic buffered saline. Cells were then exposed to hypo- and hypertonic test solutions for 15 min before being returned to isotonic conditions. Test solutions were added by washing each well three times and the cells were returned to isotonic conditions in a similar manner by washing 3 times with isotonic buffer. Cells were allowed to equilibrate with the final isotonic buffer for at least 10 min before the buffer was removed, replaced with cell culture medium, and the plate was returned to the incubator. As a control, a subset of the wells was subjected to the same wash procedures, but using isotonic solution only. The total time each plate remained out of culture conditions was less than 1 hr. A final viability assessment was performed after the cells had been in culture for 24±2 hr.

The resulting viability data were used to estimate the osmotic tolerance limits as follows. Fluorescence measurements taken 24 hr after exposure to test solutions were normalized to the average initial fluorescence measurement for the same well. This allowed us to account for potential differences in the initial cell density. The resulting normalized fluorescence was then further normalized to the average fluorescence of the control wells on the same plate that had been exposed to isotonic conditions. We call the resulting quantity after both of these normalizations the “cell yield” and estimated the osmotic tolerance limits by fitting the cell yield data with a 3-parameter logistic model:

$$Y=A+\frac{1-A}{1+{\left(\Delta M/B\right)}^{C}},$$ (2)

where *Y* is the cell yield, *ΔM* is the absolute value of the deviation in osmolality from isotonic conditions, and *A*, *B*, and *C* are constants. Best-fit values of *A*, *B*, and *C* were determined separately for hypotonic and hypertonic regimes using a least-squares Levenberg-Marquardt nonlinear fitting algorithm implemented in MATLAB.

### CPA Cytotoxicity Experiments

Our general approach to determine the toxicity of glycerol solutions consisted of assessing the initial viability using the PrestoBlue assay (as described above), equilibrating cells with glycerol solution, and then assessing the final cell viability after a 24 hr recovery period in culture. To decouple toxicity from osmotic damage, glycerol was added and removed in multiple steps (as necessary) to avoid damaging changes in cell volume. These multistep procedures were designed using membrane permeability data from our previous study [32] and are described in detail in S1 Supporting Information. Before exposure to glycerol solutions, the cells were equilibrated to the test temperature by placing the 96 well plate on a custom-made copper block, which was temperature controlled using a circulating water bath. Test solutions were also equilibrated to the test temperatures by placing solutions in a water bath for at least an hour before initiating experiments. After the cells had been exposed to the glycerol solutions, cell culture medium was added to each well, and the plate was returned to the incubator. For each change in solution composition, wells were washed 3 times. The total time each plate remained out of culture conditions was less than 1.5 hrs. The final viability was measured using the PrestoBlue assay at 24 ± 2 hr after cells had been returned to culture.

### Analysis of Cytotoxicity Data

The initial and final fluorescence values from the PrestoBlue assay were used to calculate the cell yield, as described above. To determine the rate of cytotoxicity, cell yields for different CPA exposure times were fit using a first-order cell death model:

dN/dt=−kN, (3)

where *N* is the number of viable cells and *k* is the rate of cell death due to cytotoxicity. The cell death rate was assumed to vary with intracellular CPA concentration. For multistep CPA addition and removal procedures cytotoxicity was modeled using distinct rates corresponding to the equilibrium intracellular CPA concentration in each step. It was assumed that cells reached the equilibrium concentration nearly instantly, relative to the duration of the time step. This assumption is justified by membrane transport model predictions, which show that the cells approach CPA concentration equilibration on the order of seconds [32]. To illustrate our parameter identification strategy, consider a two-step CPA addition process and a two-step CPA removal process. To prevent excessive shrinkage during CPA addition, the cells are first exposed to an intermediate CPA concentration *M*_s1_ for a period *t*_1 –_ *t*_0_ before exposure to the peak CPA concentration *M*_s2_ for a period *t*_2_–*t*_1_. To prevent excessive swelling during CPA removal, the CPA concentration is decreased to *M*_s1_ for a period *t*_3_–*t*_2_, and finally the cells are placed in isotonic buffer containing no CPA. The cytotoxicity rates during exposure to solutions with concentrations *M*_s1_ and *M*_s2_ are defined as *k*_1_ and *k*_2_, respectively. The first-order cell death model results in the following expressions for the relative number of viable cells in each step:

$$N_{1}/N_{0}=\text{exp}\left[-k_{1}\left(t_{1}-t_{0}\right)\right],$$ (4)

$$N_{2}/N_{1}=\text{exp}\left[-k_{2}\left(t_{2}-t_{1}\right)\right],$$ (5)

$$N_{3}/N_{2}=\text{exp}\left[-k_{1}\left(t_{3}-t_{2}\right)\right],$$ (6)

where *N*_0_, *N*_1_, *N*_2_, and *N*_3_ are the numbers of viable cells at the time points *t*_0_, *t*_1_, *t*_2_, and *t*_3_, respectively, and noting that the concentration during time *t*_2_ < t < *t*_3_ is *M*_s1_, the concentration associated with rate *k*_1_. Experimentally, we are able to measure the overall cell yield associated with the complete procedure:

$$Y=N_{3}/N_{0}.$$ (7)

To isolate the effects of exposure to the peak CPA concentration, Eqs 4–6 were combined to obtain an adjusted cell yield

$$\tilde{Y}=N_{2}/N_{1}=\left(N_{3}/N_{0}\right)\left(N_{2}/N_{3}\right)\left(N_{0}/N_{1}\right)=Y\text{exp}\left[k_{1}\left(t_{1}-t_{0}+t_{3}-t_{2}\right)\right].$$ (8)

The adjusted cell yield $\tilde{Y}$ can be calculated given the rate constant *k*_1_, which can be determined from experiments involving single step addition and removal of the relatively low CPA concentration *M*_s1_. The rate constant *k*_2_ associated with exposure to the peak CPA concentration *M*_s2_ can then be determined by fitting the adjusted cell yield to Eq 5. The rate of cytotoxicity for the peak CPA concentration can also be determined in this way for 3-step, 4-step, or more extensive addition and removal procedures.

The concentration dependence of the cytotoxicity death rate *k* was assumed to follow a power law [30], and the temperature dependence was assumed to follow an Arrhenius model, yielding the combined model

$$k=k_{\infty }\text{exp}\left(-E_{\text{a}}/RT\right){\left(M_{\text{s}}^{\text{i}}\right)}^{\alpha },$$ (9)

where *R* is the ideal gas constant, *T* is the temperature, $M_{\text{s}}^{\text{i}}$ is the molality of intracellular CPA and *k*_∞_, *E*_a_ and *α* are best-fit constants representing the toxicity rate at infinite temperature, the activation energy for toxicity and the concentration-dependence of the toxicity rate, respectively. The toxicity cost function was defined as the integral of this concentration- and temperature-dependent toxicity rate over the duration of the CPA addition or removal process:

$$J=\int_{0}^{t_{\text{f}}}k_{\infty }\text{exp}\left(-E_{\text{a}}/RT\right){\left(M_{\text{s}}^{\text{i}}\right)}^{\alpha }dt,$$ (10)

where *J* is the toxicity cost and *t*_f_ is the protocol duration. This toxicity cost can be expressed in terms of the overall cell yield using the first-order cell death model:

$$N_{\text{f}}/N_{0}=\text{exp}\left(-J\right).$$ (11)

Thus, minimizing the toxicity cost is equivalent to maximizing cell yield.

### Design of Toxicity-Minimized Procedures

To design minimally-toxic CPA addition and removal procedures, we used the mathematical optimization strategy described in our previous studies [30, 31]. The basic approach is to use predictions of cell membrane mass transport to determine the toxicity cost and cell volume excursions for each candidate procedure and to iteratively vary the procedural details (e.g., concentrations, exposure times) to minimize the toxicity cost subject to the osmotic tolerance limits as constraints on the cell volume. In particular, the toxicity cost function (Eq 10) was calculated using the predicted intracellular CPA concentration $M_{\text{s}}^{\text{i}}$ from the membrane transport model [32]

$$\begin{array}{l} \frac{d{\bar{V}}_{\text{w}}}{dt}=\frac{L_{\text{p}}A}{V_{\text{w0}}}\rho_{\text{w}}RT\left(M_{\text{s}}^{\text{i}}+M_{\text{n}}^{\text{i}}-M_{\text{s}}^{\text{e}}-M_{\text{n}}^{\text{e}}\right),\\ \frac{d{\bar{V}}_{\text{s}}}{dt}=\frac{P_{\text{s}}A}{V_{\text{w0}}}\nu_{\text{s}}\rho_{\text{w}}\left(M_{\text{s}}^{\text{e}}-M_{\text{s}}^{\text{i}}\right),\\ M_{\text{n}}^{\text{i}}=\frac{M_{0}}{{\bar{V}}_{\text{w}}},\\ M_{\text{s}}^{\text{i}}=\frac{{\bar{V}}_{\text{s}}}{\nu_{\text{s}}\rho_{\text{w}}{\bar{V}}_{\text{w}}}, \end{array}$$ (12)

where ${\bar{V}}_{\text{w}}$ and ${\bar{V}}_{\text{s}}$ are the intracellular volumes of water and CPA (normalized to the isotonic water volume), subscripts s and n describe CPA and nonpermeating solute, respectively, superscripts i and e describe intra- and extracellular quantities, the membrane permeability parameters *L*_p_ *A*/*V*_w0_ and *P*_s_ *A*/*V*_w0_ are taken from our previous study [32], *ρ*_w_ = 1 kg/L is the density of pure water, *ν*_s_ = 0.071 L/mol is the molar volume of glycerol and *M*_0_ = 0.3 Osm/kg is the isotonic solute osmolality. These membrane transport predictions were also used to apply the cell volume constraints ${\bar{V}}_{\text{low}}\leq {\bar{V}}_{\text{w}}+{\bar{V}}_{\text{s}}\leq {\bar{V}}_{\text{high}},$ where ${\bar{V}}_{\text{low}}$ and ${\bar{V}}_{\text{high}}$ are the lower and upper osmotic tolerance limits.

For simplicity, we chose to optimize CPA addition and removal procedures consisting of two step-changes in solution composition, and we limited ourselves to a single CPA (i.e., glycerol) instead of a combination of more than one CPA. Thus, the parameters to be optimized included the duration, temperature (*T*), glycerol concentration ($M_{\text{s}}^{\text{e}}$) and nonpermeating solute concentration ($M_{\text{n}}^{\text{e}}$) for each step of the procedure. A glycerol concentration of 17 molal was used as the target concentration after CPA addition. Following our previous work, this target glycerol concentration was the only end point condition and an additional volume condition was not imposed [31]. To further ensure that the resulting optimized procedures would be experimentally practical, each step was limited to a duration of at least 1 min and the temperature was constrained to the range 4°C ≤ *T* ≤ 37°C. Finally, we set a lower bound of 80 mOsm/kg for the nonpermeating solute concentration $M_{\text{n}}^{\text{e}}$ to avoid potential problems with ionic imbalances and to enable the solutions to be pH buffered.

### Evaluation of Toxicity-Minimized Procedures

Cells were subjected to the optimized CPA addition and removal procedures by physically moving 12 mm coverslips to temperature-controlled solutions in 3 cm petri dishes. Temperature control was achieved with a hot plate (37°C) or an ice bath (4°C). Coverslips were immersed in each solution for the specified time and moved between petri dishes using fine-point tweezers. After the final removal step, coverslips were returned to a 24-well plate, covered with medium, and returned to culture. Viability was determined 24 ± 2 hr after being returned to culture using the PrestoBlue assay as described above. For comparison, samples were also subjected to single step and conventional multistep CPA addition and removal procedures. Solution compositions, exposure times and predicted cell volume changes for these procedures are provided in S1 Supporting Information.

### Vitrification

Cells exposed to toxicity-minimized procedures were tested for the ability to vitrify using a temperature-controlled cryostage. Cells cultured on 12 mm coverslips were equilibrated with the vitrification solution and immediately transferred to the stage of an FDCS 196 cryostage (Linkham, Surrey, UK) that had been pre-cooled to a temperature of 4°C. Time-lapse imaging was initiated using a Phantom v7.1 camera (Vision Research, Wayne, NJ). Controlled-cooling was then initiated at the fastest programmable cooling rate, theoretically 130°C/min, to a final temperature of -150°C. The actual cooling rate varied with time, but averaged about 50°C/min. Cells were held at -150°C for 2 min before warming was initiated at 130°C/min.

### Statistical Analysis

Cell yield data was analyzed using ANOVA and Tukey-Kramer HSD tests. Differences were considered to be significant at a confidence level of 95%. All analysis was performed using Statgraphics statistical software (Statpoint Technologies, Inc., Warrenton, VA, USA, Version 17.1.06).

## Results

### Osmotic Tolerance Limits

**Fig 1:**
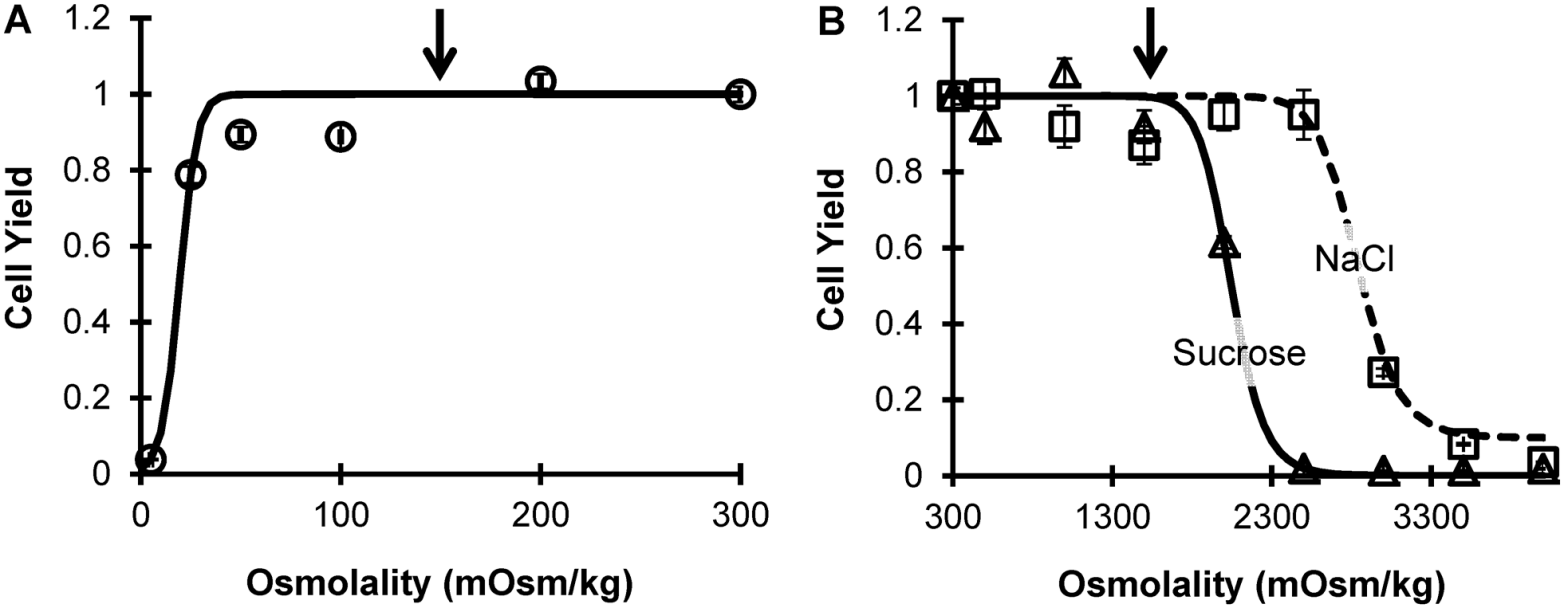
Cell yield after 15 min exposure to hypotonic (A) or hypertonic conditions (B). Hypertonic solutions were prepared using either sucrose or NaCl, as indicated. Lines show best-fit logistic models (Eq 2). Arrows indicate the osmotic tolerance limits used in our mathematical optimization algorithm.

Fig 1 shows the cell yield after 15 min exposure to a range of hypo- and hypertonic conditions. As expected, the cell yield decreased as the deviation from isotonic conditions increased. Cultured bovine pulmonary artery endothelial cells were surprisingly resistant to hypotonic conditions (Fig 1A), exhibiting a cell yield of 79 ± 2% after exposure to a solution containing only 25 mOsm/kg solutes. Nonetheless, the cell yield decreased to nearly zero after exposure to pure water. To define the upper volume osmotic tolerance limit for use in our optimization algorithm, we assumed that the cells could tolerate the swelling induced by exposure to concentrations above 150 mOsm/kg (arrow in Fig 1A). This corresponds to a limit on the osmotically active cell volume of twice the isotonic volume (i.e., ${\bar{V}}_{\text{high}}=2$). To determine the lower volume osmotic tolerance limit, the cells were exposed to hypertonic solutions prepared using either sucrose or NaCl (Fig 1B). Hypertonic sucrose solutions were more damaging than hypertonic NaCl solutions. As a conservative estimate for the lower volume osmotic tolerance limit, we assumed the cells could tolerate the shrinkage induced by exposure to concentrations up to 1500 mOsm/kg (arrow in Fig 1B), which corresponds to a volume limit of 20% of the isotonic osmotically active volume (i.e., ${\bar{V}}_{\text{low}}=0.2$). These upper and lower volume limits were used as constraints in the mathematical optimization algorithm.

### CPA Cytotoxicity

**Fig 2:**
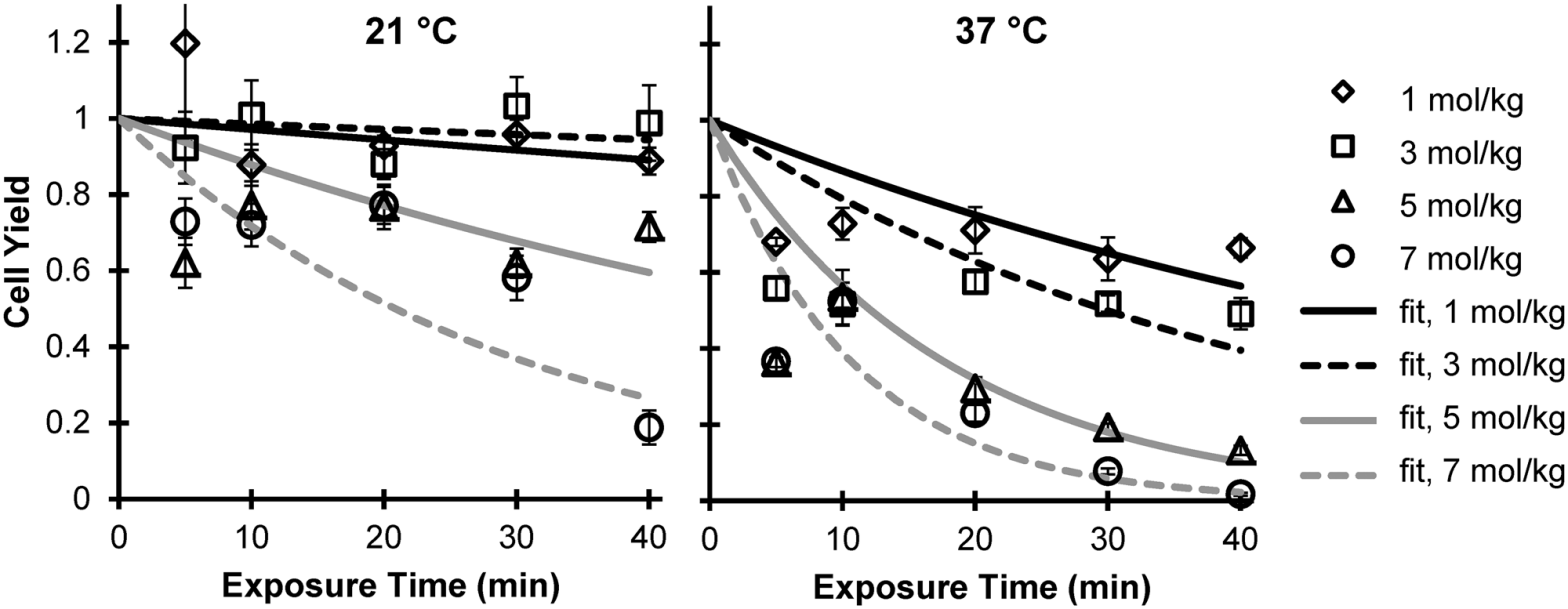
Cytotoxicity of glycerol solutions at 21°C and 37°C. Lines show best-fit exponential decay models.

The concentration- and temperature-dependence of glycerol toxicity was determined by exposing the cells to a range of glycerol concentrations at two temperatures, 21°C and 37°C. To decouple toxicity and osmotic damage, the cells were brought to the peak glycerol concentration using multiple steps as necessary (see S1 Supporting Information). Fig 2 depicts the time-dependent cell yields for glycerol exposures at 21°C and 37°C. In general, cell yield decreased as glycerol exposure time increased, as glycerol concentration increased and as temperature increased. Indeed, statistical analysis by 3-way ANOVA revealed that all of these factors (i.e., exposure time, concentration and temperature) had statistically significant effects on the cell yield (p < 0.0001). An exponential decay model was fit to the data to determine a cytotoxicity rate constant *k* for each glycerol concentration and temperature. Although the data has high variability and deviates from the exponential decay model in some cases, the results show that the rate of cell death increases with both glycerol concentration and temperature.

**Fig 3:**
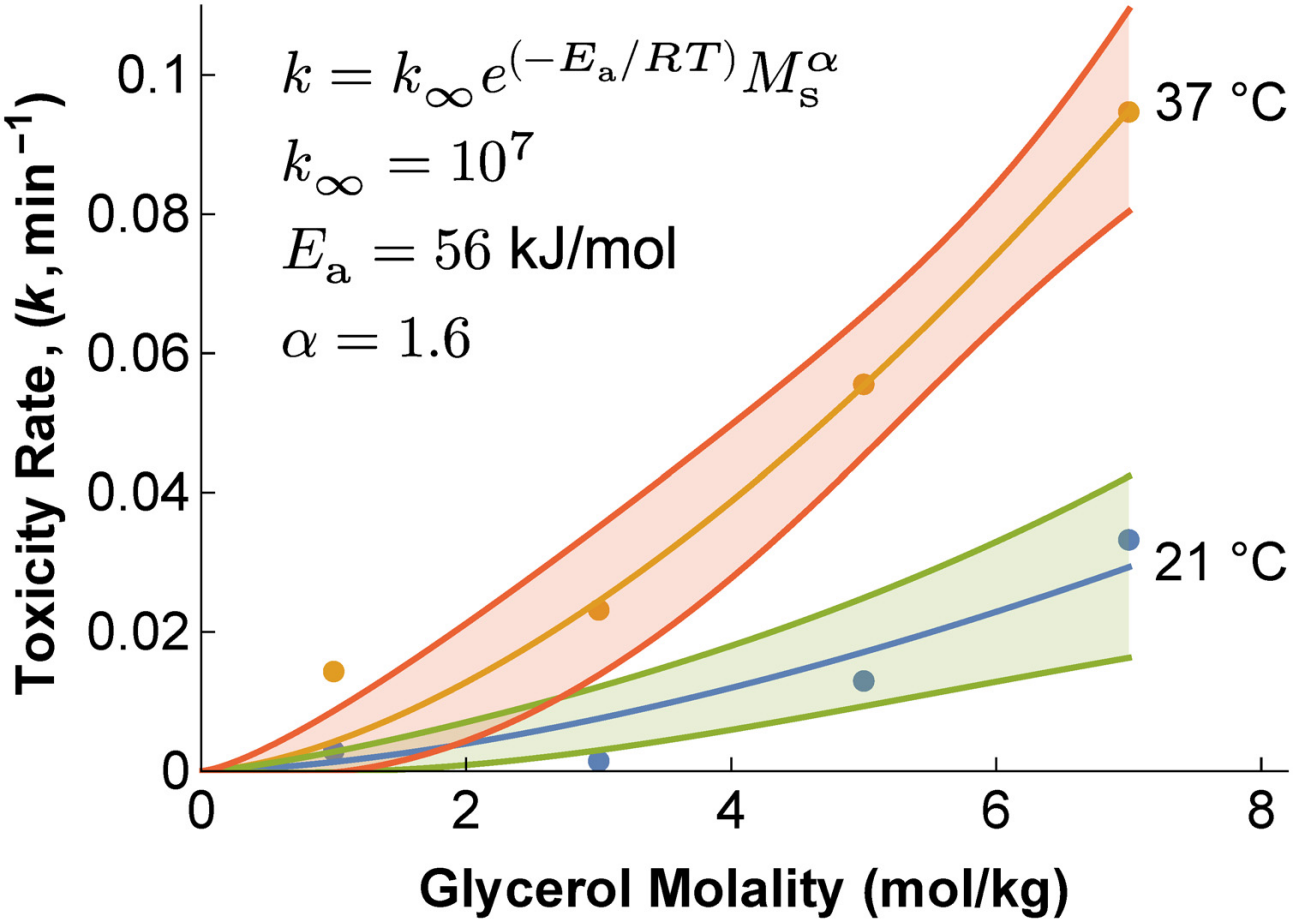
Effect of concentration and temperature on the toxicity rate constant *k*. Lines represent predictions of the concentration- and temperature-dependent toxicity rate model (Eq 9), and the shaded bands represent 95% confidence intervals. Best-fit model parameters are shown on the plot.

These trends are even more noticeable when the best-fit toxicity rate constants are plotted as a function of concentration, as shown in Fig 3. The toxicity rate is higher at 37°C than 21°C, and the toxicity rate increases with glycerol concentration. The toxicity rate data shown in Fig 3 was fit using Eq 9, resulting in an activation energy *E*_a_ = 56 ± 8 kJ/mol, a concentration exponent *α* = 1.6 ± 0.2, and a coefficient *k*_*∞*_ = 10^7^ ± 10^7^. This best-fit model was inserted into Eq 10 to define the toxicity cost function for use in the mathematical optimization algorithm.

### Optimized Procedure with Temperature Fixed at 37°C

**Fig 4:**
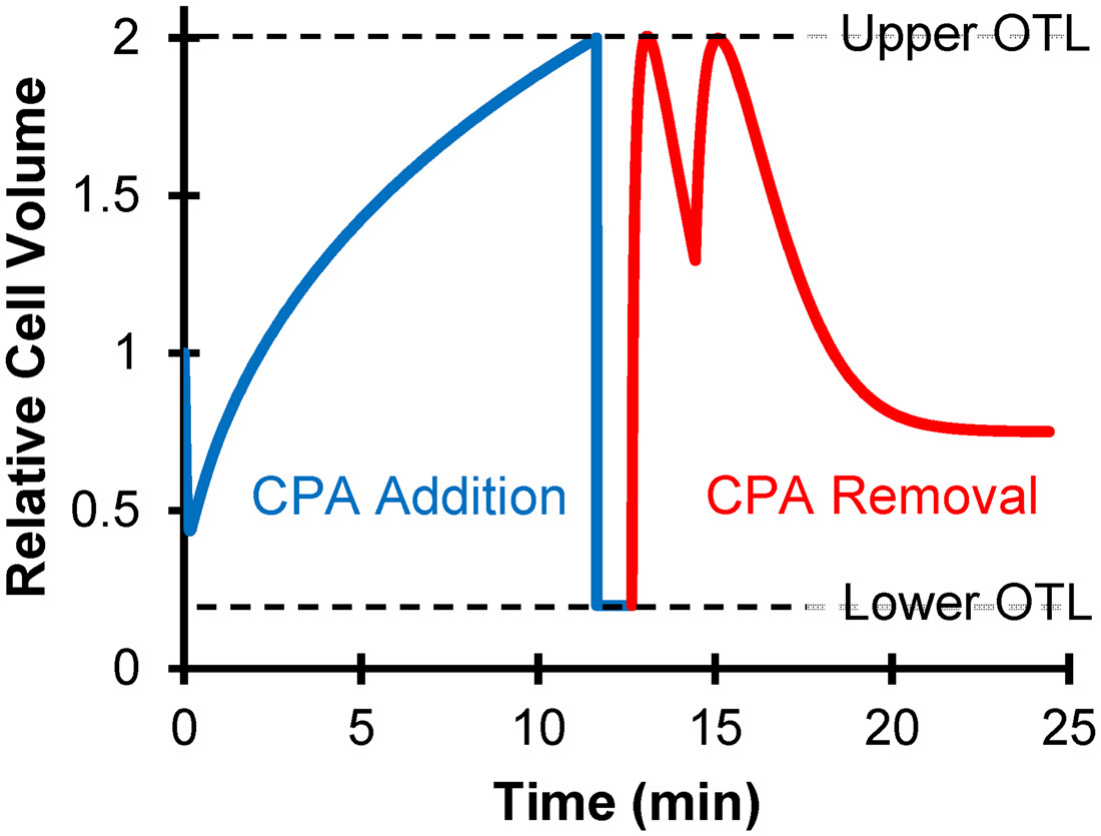
Predicted cell volume excursions for the mathematically optimized procedure in Table 1. Temperature was fixed at 37°C in the optimization algorithm. Horizontal dashed lines show the osmotic tolerance limits.

We first used the mathematical optimization algorithm to design two-step procedures for addition and removal of glycerol at a constant temperature of 37°C. To ensure sufficient glycerol loading for vitrification, the goal state at the end of CPA addition was set to an intracellular glycerol concentration of 17 molal. The resulting toxicity-minimized procedure is shown in Table 1, and the corresponding cell volume predictions are shown in Fig 4. Notably, the mathematically optimized procedure calls for a hypotonic concentration of nonpermeating solutes during the first CPA loading step, which is predicted to result in cell swelling to the maximum osmotic tolerance limit. The second step involves exposure to a 17 molal glycerol solution containing a hypertonic concentration of nonpermeating solutes; this is predicted to cause cell shrinkage to the minimum osmotic tolerance limit, thus concentrating the intracellular CPA that had been loaded in the first step. Using Eq 11, the predicted cell yield after CPA addition is 70%. The optimized CPA removal process involves exposure to glycerol-free solutions containing a hypertonic concentration of nonpermeating solutes to prevent excessive swelling. Swelling to the maximum osmotic tolerance limit during CPA removal dilutes intracellular glycerol, thus reducing toxicity. As a result, the cell yield associated with the CPA removal process (99%) is much higher than the predicted cell yield for CPA addition. The overall cell yield can be calculated by multiplying the yields for CPA addition (70%) and CPA removal (99%), resulting in 69%.

**Table 1:** Mathematically optimized procedures for addition and removal of 17 molal glycerol at 37°C.

	Step	Glycerol (mol/kg)	Nonpermeating solute (Osm/kg)	Time (min)	Predicted Cell Yield
CPA Addition	1	0.83	0.08	12	70%
	2	17	3.3	1	
CPA Removal*	1	0	0.8	2	99%
	2	0	0.4	10	

* Several CPA removal procedures had nearly identical toxicity costs, making it difficult to identify the global optimum. The given procedure was selected from this group for experimental expediency.

**Fig 5:**
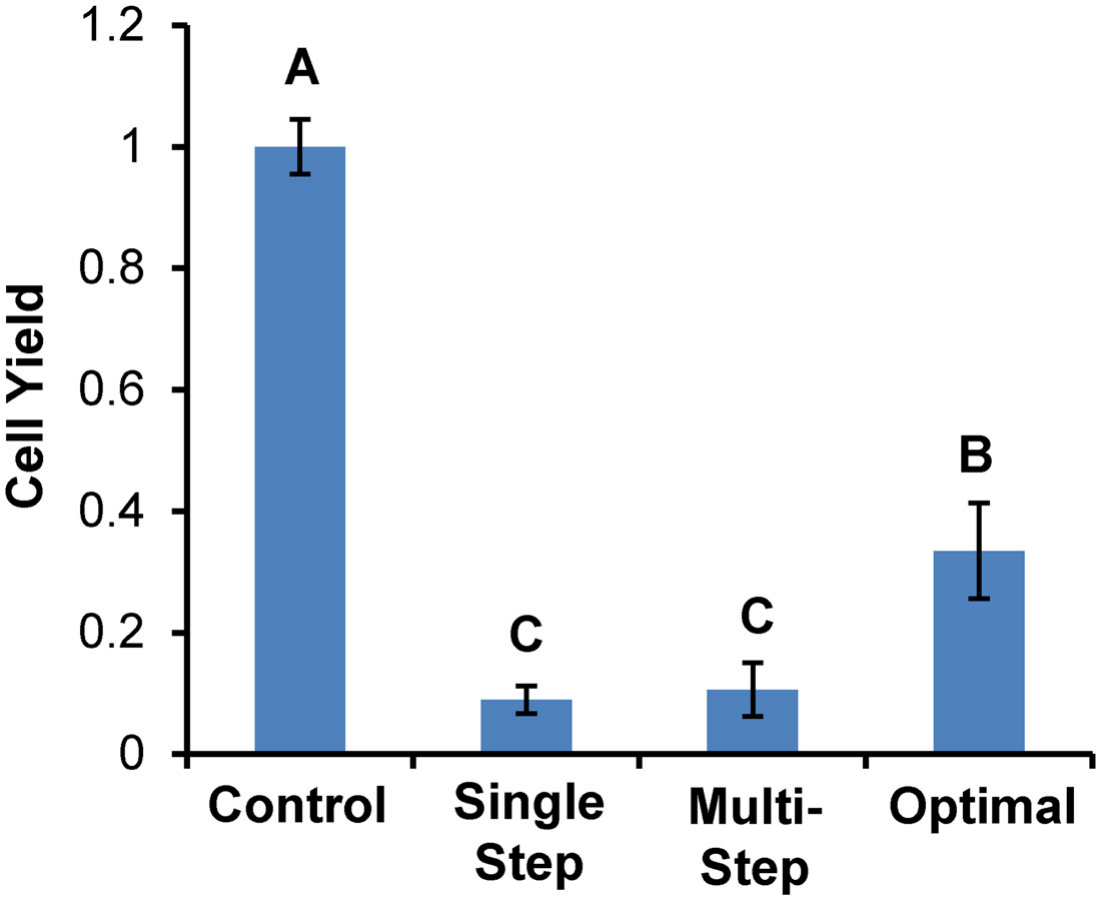
Cell yield for the mathematically optimized procedure in Table 1. For comparison, the results for single step and conventional multistep procedures are also shown. All experiments were carried out at 37°C. Bars marked with distinct letters are significantly different (p < 0.05).

Fig 5 compares the experimentally measured cell yield for the mathematically optimized procedure to the results for single-step and conventional multistep CPA addition and removal procedures. The cell yield for the toxicity-minimized procedure (33% ± 8%) was about two-fold lower than the predicted value (see Table 1). Nonetheless, it was significantly higher than the cell yield for the single-step (9% ± 2%) and conventional multistep (11% ± 4%) procedures (*p* < 0.04). All of the procedures for addition and removal of 17 molal glycerol resulted in a significant reduction in cell yield compared with the control (*p* < 0.0001).

### Optimized Procedure with Temperature Constrained between 4°C and 37°C

Our model of the temperature dependence of glycerol toxicity allowed us to include the temperature of each step as a control variable in our optimization in addition to the step duration and solute concentrations. The resulting mathematically optimized procedure was very similar to that shown in Table 1 and Fig 4, except that the temperature in the second addition step was 4°C instead of 37°C. There were also minor differences in the optimal concentrations, as detailed in Table 2. This temperature-optimized CPA equilibration process is predicted to result in an overall cell yield of 94%, substantially higher than that predicted when the procedure was optimized at a fixed temperature of 37°C.

**Table 2:** Mathematically optimized procedures for addition and removal of 17 molal glycerol with temperature constrained between 4°C and 37°C.

	Step	Glycerol (mol/kg)	Nonpermeating solute (Osm/kg)	Time (min)	Temperature (°C)	Predicted Cell Yield
CPA Addition	1	0.87	0.08	12	37	95%
	2	20	0.2	1	4	
CPA Removal*	1	0	0.8	2	37	99%
	2	0	0.4	10	37	

* Several CPA removal procedures had nearly identical toxicity costs, making it difficult to identify the global optimum. The given procedure was selected from this group for experimental expediency.

**Fig 6:**
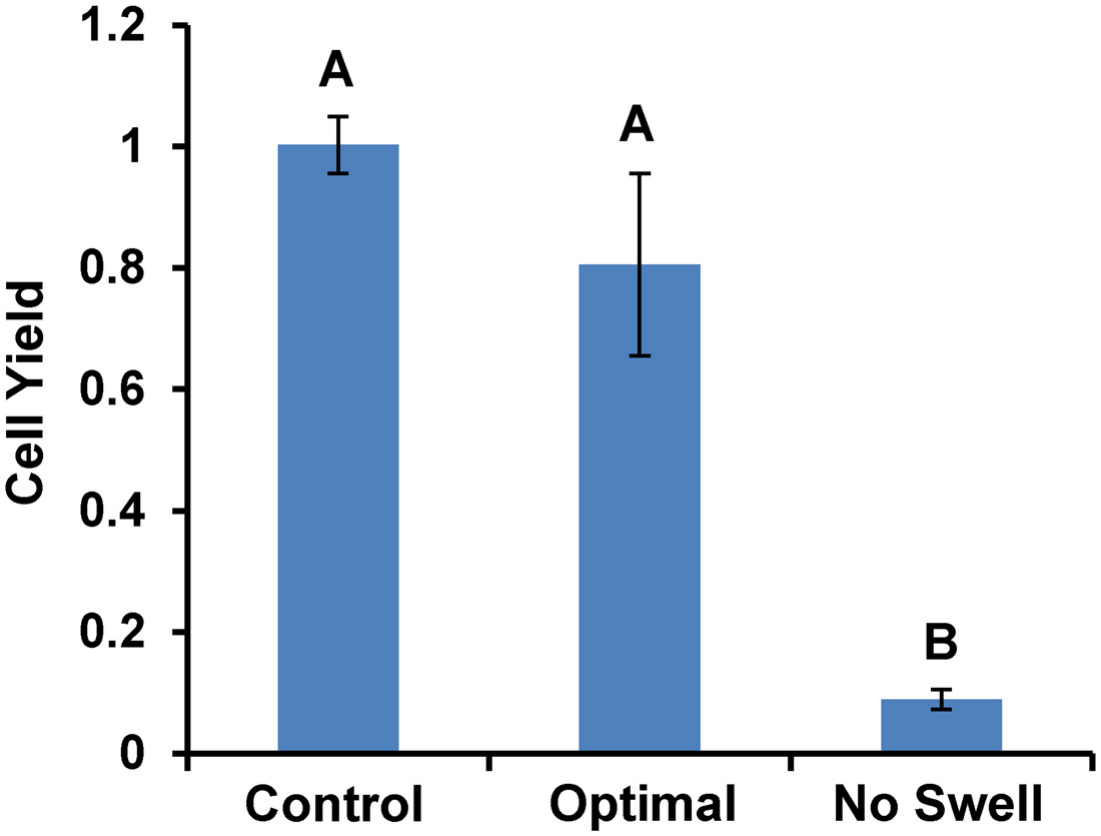
Effect of temperature and cell swelling on cell yield. The bar labelled “optimal” refers to the procedure in Table 1, with the first CPA addition step carried out at 37°C and the second at 4°C. The procedure labeled “no swell” was identical, except that an isotonic concentration of nonpermeating solutes was used in the first loading step, instead of the hypotonic concentration shown in Table 1. Bars marked with distinct letters are significantly different (p < 0.05).

Fig 6 shows experimental results for the temperature-optimized procedure. For convenience and to facilitate direct comparisons with the single temperature protocols, we chose to reuse the solutions from our previous experiments (see Table 1), but carry out the second CPA addition step at 4°C instead of 37°C. This minor change in the optimized solution compositions (compared with Table 2) is predicted to have a negligible effect on cell yield. As shown in Fig 6, the temperature-optimized procedure resulted in a much improved cell yield of 81% ± 15%, a value that was statistically indistinguishable from the control.

A distinguishing feature of the mathematically optimized procedures compared with most classical procedures is the use of a hypotonic concentration of nonpermeating solute during CPA loading, which induces swelling to the upper osmotic tolerance limit. In contrast, the conventional approach for CPA loading uses an isotonic concentration of nonpermeating solute and focuses on avoiding excessive cell shrinkage. Therefore, we examined the value of the predicted optimal hypotonic loading approach by conducting a control experiment in which the hypotonic nonpermeating solute concentration in the first loading step was replaced with an isotonic concentration. This modified procedure is not expected to induce swelling. As shown in Fig 6, this modified “no swell” procedure resulted in a low cell yield of 9% ± 2%, which indicates that cell swelling during CPA addition is essential to the success of our toxicity-minimized procedures.

### Vitrification

**Fig 7:**
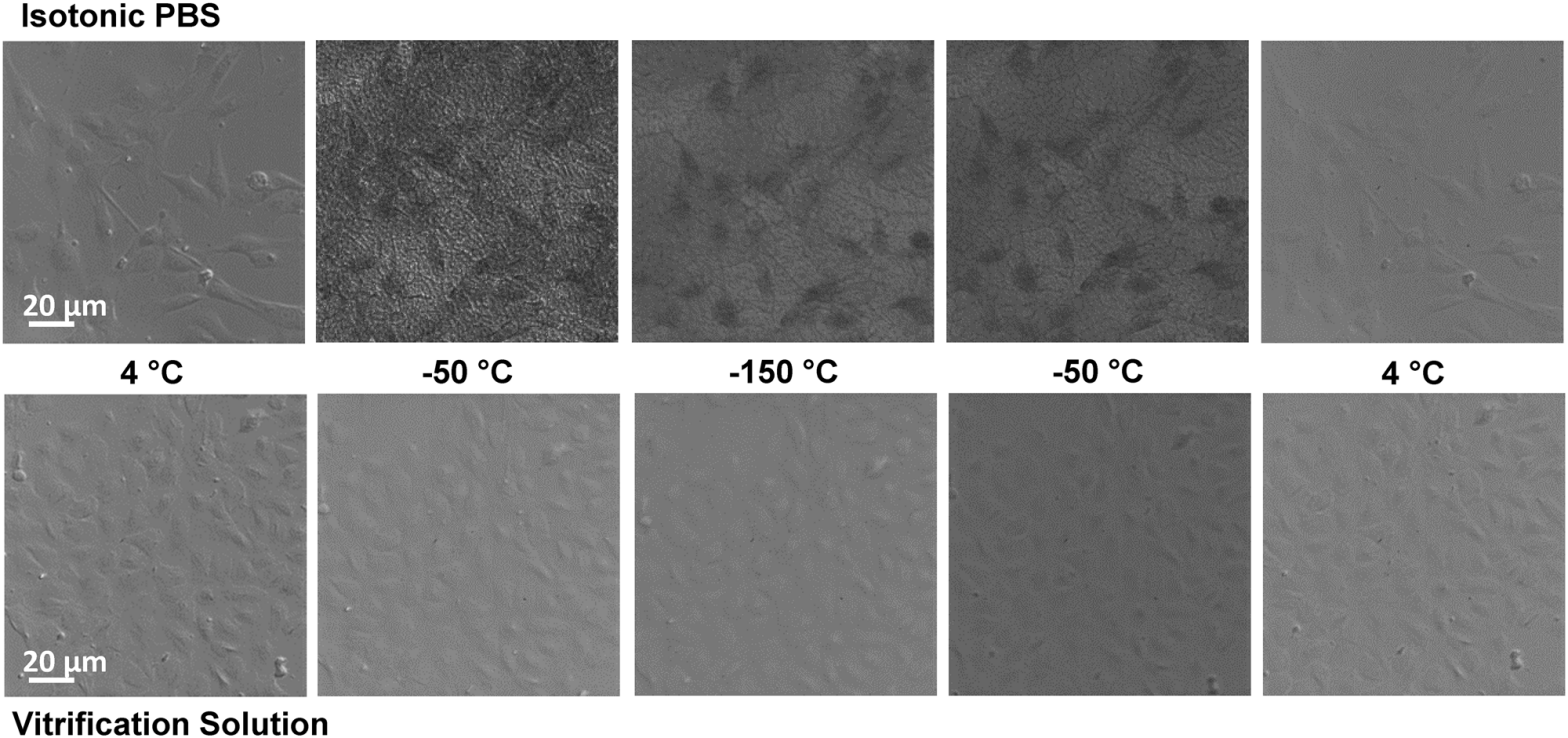
Vitrification of cultured endothelial cells. Time-lapse images during cooling and warming show clear evidence of intra- and extracellular ice formation for cells in isotonic buffer (Top), but no evidence of ice formation for cells in vitrification solution (Bottom).

To test the feasibility of vitrification with our toxicity-minimized procedures, cells were imaged while undergoing cooling and warming on a temperature-controlled cryostage. Representative images are shown in Fig 7. As cells in isotonic buffer are cooled, cell darkening appears, which is indicative of intracellular ice formation. Also, images gain a grainy appearance due to extracellular ice formation. Ice disappears as the sample warms above the melting point. For cells in the vitrification solution containing 17 molal glycerol, no evidence of intracellular or extracellular ice formation was seen in the time-lapse images, suggesting that the cells were successfully vitrified.

### Sensitivity of Mathematically Optimized Procedures to Toxicity Model Parameters

**Fig 8:**
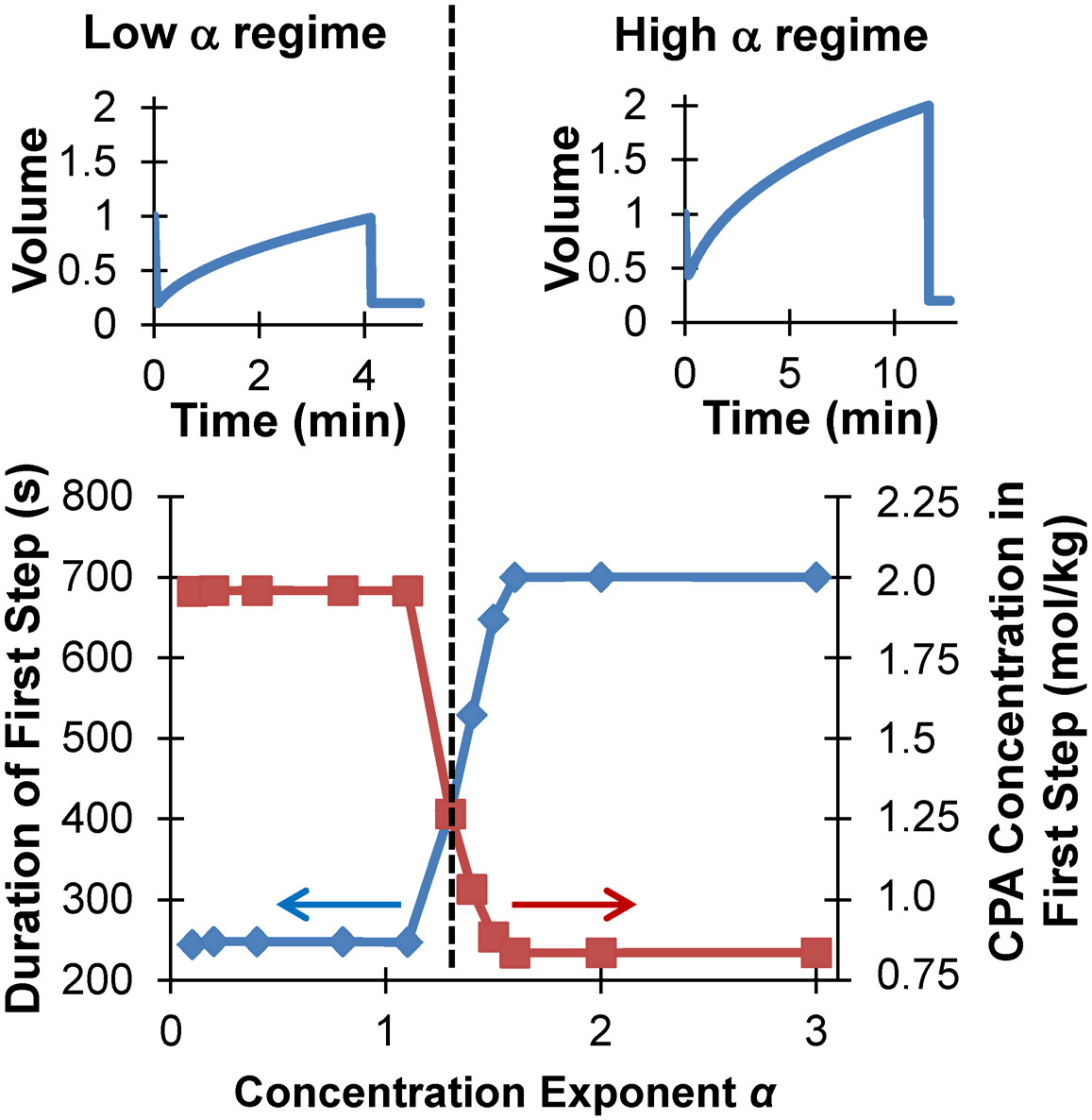
Effects of the CPA cytotoxicity model parameter *α* (see Eq 9) on mathematically optimized procedures for addition of 17 molal glycerol. While the second CPA addition step was essentially identical for all *α*-values, the conditions in the first step can be divided into low *α* and high *α* regimes. In the low *α* regime, the cells are exposed to a relatively high CPA concentration for a relatively short duration (bottom panel), resulting in shrinkage to the minimum volume limit (top left panel). In contrast, the high *α* regime involves exposure to a relatively low CPA concentration for a relatively long duration, which results in swelling to the maximum volume limit (top right panel).

Because of the considerable uncertainty in estimation of the parameters in the CPA toxicity model (Eq 9; see Figs 2 and 3), we examined the effects of varying the model parameters on the resulting mathematically optimized procedures. Our analysis focused on the effects of the concentration exponent α and the activation energy *E*_a_; the coefficient *k*_*∞*_ was not included in the sensitivity analysis because it only scales the cost and therefore has no effect on the predicted optimal protocols. Fig 8 illustrates the effects of the concentration exponent *α*. Variation of *α* around the best-fit value (*α* = 1.6) revealed two regimes within which the optimized CPA addition procedures were identical. In the high *α* regime (*α* ≥ 1.6), optimal CPA loading is achieved by exposure to a relatively low CPA concentration in a hypotonic carrier solution, which causes swelling to the maximum osmotic tolerance limit at the end of step 1 (see top right panel of Fig 8). This approach minimizes the CPA concentration (and hence the toxicity rate) during the loading process, but requires a relatively long duration. In the low *α* regime (*α* ≤ 1.1), the rate of CPA toxicity is relatively insensitive to CPA concentration. In this regime, optimal CPA loading is achieved by exposure to the highest CPA concentration that does not cause excessive cell shrinkage. Thus, the cells shrink to the minimum osmotic tolerance limit in the first step (see top left panel of Fig 8). This approach maximizes the driving force for cellular uptake of CPA and thus minimizes the time required for CPA loading. Comparison of the step durations and CPA concentrations shown in Fig 8 (bottom panel) helps to illustrate the key differences in the optimized procedures: for low *α* it is preferable to minimize CPA exposure time, even if it requires exposure to a relatively high CPA concentration, whereas for high *α* it is preferable to minimize the CPA concentration, even if it requires a longer CPA loading time.

**Fig 9:**
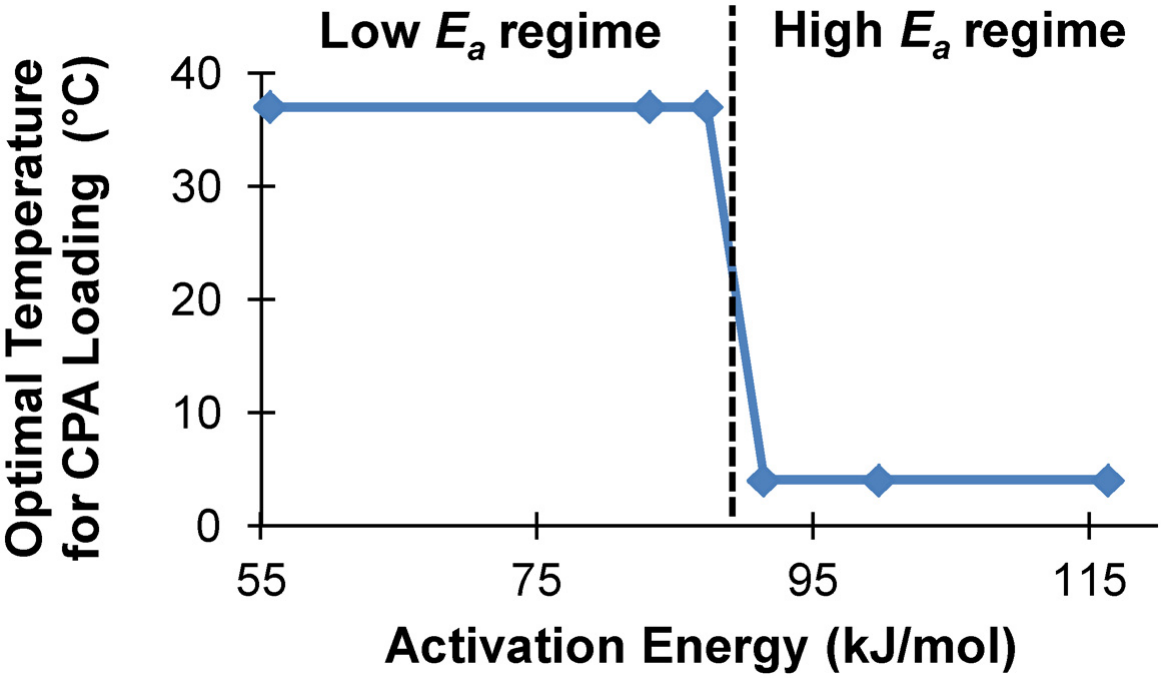
Effects of the activation energy for CPA cytotoxicity (*E*_a_, see Eq 9) on mathematically optimized procedures for addition of 17 molal glycerol. While the temperature in the second CPA addition step was 4°C for all *E*_a_ values, the temperature in the first step can be divided into low *E*_a_ and high *E*_a_ regimes. The transition between these regimes occurs at 89 kJ/mol (vertical dashed line); this value matches the activation energy for glycerol transport [32].

Fig 9 illustrates the effect of the activation energy for CPA cytotoxicity (*E*_a_ in Eq 9) on the optimal temperature for CPA loading. Once again, variation of *E*_a_ around the best-fit value (*E*_a_ = 56 kJ/mol) resulted in two different CPA loading regimes. In the high *E*_a_ regime (*E*_a_≥ 91 kJ/mol), the optimal CPA loading temperature is 4°C (i.e., the minimum temperature considered in the optimization algorithm). In this case, the loading process is relatively long (~12h), but it is predicted to be less damaging because CPA cytotoxicity is highly temperature dependent and much slower at lower temperatures. In the low *E*_a_ regime (*E*_a_ ≤ 87 kJ/mol), the optimal temperature for CPA loading is 37°C (i.e., the highest temperature considered in the optimization algorithm). This loading process is much faster (~12 min) because glycerol transport is faster at high temperatures. The transition between these two CPA loading regimes occurs when the activation energy for CPA cytotoxicity is ~89 kJ/mol, which is equal to the activation energy for glycerol transport across the cell membrane [32].

## Discussion

Although significant advancements in vitrification methods have been reported over the last few decades, CPA cytotoxicity remains a major limitation, especially for relatively large samples such as adherent cells, tissues and organs [14, 20]. We recently presented a new strategy for avoiding toxicity during vitrification by mathematically minimizing a CPA cytotoxicity cost function, and showed promising theoretical predictions for vitrification of human oocytes [30, 31]. In this study, we have expanded the optimization strategy to account for the effects of temperature and experimentally validated the optimized procedures using adherent endothelial cells as a model system.

It is well recognized that CPA cytotoxicity is dependent on CPA concentration and temperature [16, 21, 23]. Nonetheless, most previous strategies for rational design of CPA equilibration procedures do not account for the effects of these factors. The most common approach neglects toxicity altogether and focuses instead on avoiding osmotic damage [18]. The resulting procedures typically have multiple steps in which CPA concentrations are changed gradually to keep osmotically induced cell volume changes from exceeding tolerable limits. While this approach is effective for relatively low CPA concentrations [18], cytotoxicity can become problematic when loading the high CPA concentrations required for vitrification. Our results show that conventional multistep procedures result in 90% cell loss when loading adherent endothelial cells with 17 molal glycerol. In contrast, the mathematically optimized procedure, which avoids osmotic damage while also accounting for the effects of both CPA concentration and temperature on cytotoxicity, resulted in a cell yield of ~80%.

In the present study, we showed that bovine pulmonary artery endothelial cells were able to tolerate osmotically active volumes from 0.2 to 2 times their isosomotic volume, which corresponds to a solute osmolality range of 150 mOsm/kg to 1500 mOsm/kg. For comparison, Kashuba et al. [34] published osmotic tolerance limits for a number of mouse embryonic stem cell lines (mESC) that range from approximately 140 mOsm/kg to between 671 mOsm/kg and 1075 mOsm/kg. This lower osmolality limit, corresponding to an upper volume osmotic tolerance limit ${\bar{V}}_{\text{high}}\;=\;2.1$, is comparable to the present study, though their published recovery at 75 mOsm/kg was considerably lower than in the present study. The upper osmolality limit for mESCs corresponds to a lower volume osmotic tolerance limit of 0.28–0.45, which is slightly more restrictive than the limit for endothelial cells from the present study. However, these osmotic tolerance ranges for both cell types are large compared with sperm from a number of species. For example, human sperm only tolerate osmolalities between 240 mOsm/kg and 600 mOsm/kg, which corresponds to osmotically active volume limits of 1.2 and 0.48 [18]. For human sperm, multistep protocols are required even for 1 mol/kg CPA solutions due to the limited range that sperm can shrink and swell before damage [18]. This problem is exacerbated for higher CPA concentrations, making it technically challenging if not impossible to equilibrate sperm with the 17 molal CPA medium used in the present study. However, because of their similar osmotic tolerance, it is reasonable to expect that mESC might behave similarly to the endothelial cells from the present study.

To account for the effects of CPA concentration on toxicity, we used a rate model with a power law dependence on CPA concentration. In our previous work [30], we combined toxicity data from two published sources [21, 23] to estimate the effects of the concentration of dimethyl sulfoxide (DMSO) on the toxicity rate, resulting in a concentration exponent *α* = 1.6. In the current study, we also obtained a concentration exponent of 1.6 ± 0.2 for exposure of cultured endothelial cells to glycerol. This strong concentration dependence of the toxicity rate results in a predicted optimal procedure that minimizes CPA concentration during CPA loading. In particular, the optimized procedure involves the use of a hypotonic carrier solution in the first CPA addition step, which causes swelling to the maximum osmotic tolerance limit. The use of a hypotonic carrier solution, and the concomitant cell swelling, allows a relatively large amount of CPA to be loaded into the cells while using a relatively low CPA concentration. This is because the amount of intracellular CPA is the product of cell volume and CPA concentration. The use of a hypotonic carrier solution to induce swelling during CPA addition differs from the conventional approach of using an isotonic carrier solution. Our results clearly show that the hypotonic carrier solution is advantageous; carrying out the optimized procedure using isotonic carrier solution (while otherwise keeping the protocol the same) substantially reduced cell yield from 81% to less than 10%. This large reduction in cell yield can most likely be attributed to insufficient CPA loading in the first step, followed by excessive shrinkage upon exposure to the vitrification solution in the second step.

To account for the temperature dependence of CPA toxicity, we assumed the toxicity rate followed an Arrhenius model and obtained a best-fit activation energy of *E*_a_ = 56 ± 8 kJ/mol for the toxicity of glycerol to cultured endothelial cells. This is consistent with previous studies of the temperature dependence of CPA toxicity [16, 21, 23]. Lawson and colleagues [25] fit a similar toxicity model to their data and obtained activation energies of 36 kJ/mol and 41 kJ/mol for the toxicity of DMSO and propylene glycol, respectively, to alginate-encapsulated mouse insulinoma cells. Wang et al [23] and Elmoazzen et al [21] reported DMSO toxicity kinetics as a function of temperature but did not explicitly report the activation energy for the toxicity rate. Nonetheless, their data can be used to estimate the activation energy, resulting in values as high as 75 kJ/mol for articular cartilage [21] and as low as 10 kJ/mol for dermal fibroblasts [23]. When we allowed temperature to vary in our optimization algorithm, we found that the optimal temperature for CPA loading was 37°C (i.e., the upper bound on temperature in the optimization algorithm) and the optimal temperature for exposure the final vitrification solution was 4°C (i.e., the lower bound on temperature). This procedure resulted in a cell yield of 81%, substantially higher than the cell yield of 33% obtained when the temperature was fixed at 37°C in the optimization algorithm.

It is perhaps surprising that our mathematically optimized procedure worked so well, given the considerable variability in the glycerol toxicity data and the uncertainty involved in estimation of the parameters in the toxicity cost function. There are several possible causes for the variability in the toxicity data. In particular, the multistep procedures that were used to prevent osmotic damage increased the risk of cell loss due to extensive handling and the complexity of the procedures increased the likelihood for operator error. In addition, it was impractical to completely randomize treatments by well because this would have required the use of a single channel pipet to introduce solutions into each well individually; to carry out the complex and time sensitive multistep procedures it was necessary to use a multichannel pipet. As a result, the well plates were randomized by row. This introduces the possibility of systematic error due to uneven cell seeding density or edge effects. All of these factors may have contributed to the high variability shown in Fig 3, as well as the deviations from model predictions.

The high variability in the toxicity data motivated us to explore the sensitivity of the optimized procedures to the parameter values in the toxicity cost function. Our results show that, under the optimization conditions used in this study, there are only four possible classes of CPA equilibration procedures (see Figs 8 and 9). The optimal solution composition during CPA loading depends on the value of the concentration exponent α. When α is high, the optimal CPA loading solution contains a hypotonic concentration of nonpermeating solutes and a relatively low CPA concentration, which induces swelling to the maximum osmotic tolerance limit. On the other hand, when α is low the CPA loading process induces shrinkage to the minimum osmotic tolerance limit using a relatively high CPA concentration. The optimal temperature for CPA loading depends on the value of the activation energy for the toxicity rate. When *E*_a_ is high, it is preferable to have a long CPA loading process at a low temperature, but when *E*_a_ is low, fast CPA loading at high temperature is preferred. This finding is significant because it allows design of CPA equilibration procedures even without explicit knowledge of the CPA toxicity kinetics for the cell type of interest. For instance, in this study we examined the toxicity of glycerol at two temperatures over a range of concentrations and exposure times, resulting in 32 different conditions to be tested. The experiments were technically challenging, resulted in high variability and our results are specific to adherent bovine pulmonary artery endothelial cells. Rather than performing similar toxicity kinetics experiments for every cell type of interest, it would much more straightforward to simply experimentally evaluate the four possible classes of predicted optimal procedures.

The protocols parameterized by the exponent *α* described in this manuscript are somewhat like one proposed by Levin [35, 36], who suggests an optimal protocol is one that has minimal volume excursions. In Levin’s protocols, water volume is exchanged for CPA volume in a 1–1 ratio. This in some sense could be viewed as a compromise between the toxicity optimal approaches that we propose and test here, and the time optimal approaches described in previous studies [28, 30, 37, 38]. As described above, and in the above cited papers, the toxicity optimal protocol is that in which the cell is held at the upper osmotic tolerance limit for as much of the duration of the protocol as possible. For time optimal protocols the cell is held at the lower osmotic tolerance limit for as much of the duration of the protocol as possible. In this paper we show that the “switch” point between these two protocol categories seems to occur around *α* = 1, the mathematical proof of which is an area of our current research. Levin’s proposal is interesting as it embodies a “volume optimal” trajectory in which CPA toxicity plays a more minor role compared to a cost that may be strongly dependent on the time spent away from the cell’s isosmotic volume. Further experimentation with cost functions that distinguish between concentration and volume dependent damage is needed.

Our choice of a toxicity rate model in this and previous papers was proposed as a best-fit observational model, and not based on a constitutive model of accumulation of some quantity of toxicity damage. It would be illustrative to be able to construct a model from a more foundational theory of cell damage due to high concentrations of permeating solutes, even in generality, as the form of the model could be used when fitting the data. The present authors are unaware of such theory, but are working to mathematically justify our claims that the number of predicted optimal procedures are limited, at least under the present damage hypotheses, if not under the more general criteria that the damage accumulation function increases monotonically with time and concentration.

In fact, our approach is not the first to suggest using hyposmotic carrier media for CPA loading. For example, Meryman [39] discusses using hyposmotic carrier media for the equilibration of red blood cells with 40% glycerol in a minimal number of steps by taking advantage of the entire volume range that cells may safely tolerate. The approach defined by Meryman is a sort of “minimal step” approach, and may be optimal under the appropriate cost function—e.g., if the cost function is dependent explicitly on the number of exposure steps. In contrast, our model assumes mechanical damage is zero until the osmotic tolerance limits are reached when the mechanical damage is 100%. One can envision a model that accounts for an accumulation of damage both from the concentration dependent exposure to CPA and from either the number of rapid osmotic shifts or the duration of time away from the cells’ isosmotic volume. That the cost should include some function of these two possibilities was recently experimentally verified by Zou et al [40], whose data suggest that repeated shrink-swell cycles cause cell leakage and a decrease in steady state volumes and a related increase in osmotic fragility.

In this manuscript we validated a new modeling approach for designing minimally toxic CPA equilibration procedures for vitrification of bovine pulmonary artery endothelial cells. We expect that with the requisite biophysical parameters of other plated cell lines, more robust and successful cryopreservation protocols can be developed. However, in the present study, our protocol design was limited to vitrification solutions containing a single permeating CPA. While successful vitreous cryopreservation has been reported for single-CPA solutions (e.g., [41]), it is more common to use CPA cocktails [14, 15, 20, 26], and there is evidence that the use of multiple CPAs can reduce overall toxicity [20, 42]. To extend our optimization approach for use with CPA cocktails it will be necessary to develop a toxicity cost function that accounts for the effects of each individual CPA, as well as the interactions between them. The development of this model will require systematic investigation of cytotoxicity kinetics for combinations of CPAs over a range of concentrations.

CPA toxicity is arguably the most significant limiting factor in tissue and organ cryopreservation [20] and our approach provides design principles that may be used in optimizing CPA equilibration strategies that minimize toxicity. Tissues present new challenges, however. First, tissues may have multiple cell types that have differential membrane transport kinetics, toxicity rates or osmotic tolerance characteristics. Additionally, these three dimensional structures require more complicated transport models and may require models that account for biomechanical effects [43] or cell-cell, cell-interstitium, and interstitial transport [44]. Once a suitable transport model is determined, a cost function must be defined that accounts for the chemical toxicity and osmotic damage experienced by each cell in the tissue, as well as the potential effects of tissue level mechanical stresses. Finally, the transport model and the cost function must be used to determine optimal boundary controls. One exciting potential application is the cryopreservation of organ-on-a-chip systems, which are self-contained microfluidic-based systems that are designed to replicate the behavior of whole organs, including the lung [45], heart [46], or kidney [47]. Successful cryopreservation of these systems would facilitate supply for worldwide distribution. Organs and vascularized tissue allografts present further challenges as transport models are complicated by the vascular structure, that on one hand can be used for perfusion purposes and on another can be impinged by osmotically induced volume changes. Nevertheless, the principles proposed in this manuscript remain the same, namely, that models of CPA induced cytotoxicity coupled with models of CPA transport can play important roles in guiding optimal CPA equilibration strategies in cells, tissues, and even organs.

## Conclusions

In this study we have accomplished several principal goals. First is the experimental validation of a CPA toxicity cost function we previously proposed, as well as its extension to a temperature dependent model. Second is demonstration that this cost function can be used for rational design of minimally toxic CPA equilibration procedures for vitrification of adherent endothelial cells. Using the mathematically optimized procedures, we were able to expose the cells to a 55% v/v glycerol solution and retain approximately 80% cell yield, whereas conventional multistep techniques resulted in only 10% cell yield. Third, we demonstrated the generality of the optimized procedures by theoretically examining the effects of varying the parameter values in the toxicity cost function; the resulting optimized procedures can be divided into only four regimes, highlighting the potential for extension of the optimization approach to other cell types without the need for time consuming experiments to develop a toxicity cost function specific to the cell type of interest. Application of this approach to valuable cultured cell types such as human embryonic stem cells may save considerable time and money by circumventing the need for cell isolation before cryopreservation and replating after cryopreservation. Our results also pave the way for further refinement and extension of the optimization approach to more complex systems such as tissues and organs.

## Supporting Information

S1 Supporting Information Additional information about the multistep CPA equilibration procedures used in the cytotoxicity studies, as well as the single-step and conventional multistep methods used for addition and removal of 17 molal glycerol solutions. (PDF)
