# Supplementary material for: Toxicity Minimized Cryoprotectant Addition and Removal Procedures for Adherent Endothelial Cells
Source: PLoS One. 2015 Nov 25;10(11):e0142828. doi: 10.1371/journal.pone.0142828 (PMC4659675; doi:10.1371/journal.pone.0142828)
Supplement: S1 Supporting Information — (PDF) [file pone.0142828.s001.pdf]

SUPPORTING INFORMATION

Toxicity Minimized Cryoprotectant Addition and Removal Procedures for Adherent Endothelial Cells

Allyson Fry Davidson, Cameron Glasscock, Danielle R. McClanahan, James D. Benson and Adam Z. Higgins

Design of Multistep CPA Addition and Removal Procedures for CPA Cytotoxicity Studies

To decouple cell damage caused by osmotically induced cell volume changes and CPA-induced cytotoxicity, we designed multistep procedures for addition and removal of CPA that were predicted to maintain cell volumes within the osmotic tolerance limits. These predictions were made based on the cell membrane permeability data published in our previous study [1]. Tables S1 and S2 provide the exposure times and solution compositions for these multistep procedures, and Fig. S1 shows the corresponding cell volume predictions. We designed procedures for equilibration of the cells with glycerol concentrations of 1, 3, 5 and 7 molal. The nonpermeating solute concentration in these glycerol solutions was selected to ensure that the cells would equilibrate at their isotonic volume. Because glycerol occupies some intracellular volume, the nonpermeating solute concentration required for equilibration at the isotonic volume increased as the glycerol concentration increased (Table S1). Specifically, the nonpermeating solute concentration can be calculated as follows

$$M_n = M_0\left(1+\nu_s M_s\right),$$

where M_0 is the isotonic osmolality (0.3 Osm/kg), ν_s is the molar volume of glycerol (0.071 L/mol) and M_s is the molality of glycerol. To prevent excessive swelling during removal of glycerol, 1 Osm/kg nonpermeating solute was added as needed (Table S2).

Table S1. Concentrations of glycerol (M_s, mol/kg), nonpermeating solute (M_n, Osm/kg) and exposure times (t, min) during multistep addition procedures for CPA toxicity experiments.

T (°C)	Final M_s	Step 1			Step 2			Step 3	
		M_s	M_n	t	M_s	M_n	t	M_s	M_n
21	1	1	0.32						
	3	1	0.32	4	3	0.36			
	5	1	0.32	4	3	0.36	1.5	5	0.41
	7	1	0.32	4	3	0.36	1.5	7	0.45
37	1	1	0.32						
	3	1	0.32	3	3	0.36			
	5	1	0.32	3	3	0.36	1.5	5	0.41
	7	1	0.32	3	3	0.36	1.5	7	0.45

Table S2. Concentrations of glycerol (M_s, mol/kg), nonpermeating solute (M_n, Osm/kg) and exposure times (t, min) during multistep removal procedures for CPA toxicity experiments.

T (°C)	Initial M_{CPA}	Step 1			Step 2			Step 3			Step 4	
		M_s	M_n	t	M_s	M_n	t	M_s	M_n	t	M_s	M_n
21	1	0	1	4	0	0.3						
	3	1	1	5	0	1	5	0	0.3			
	5	3	1	5	1	1	5	0	1	5	0	0.3
	7	3	1	7	1	1	10	0	1	5	0	0.3
37	1	0	1	2	0	0.3						
	3	1	1	3	0	1	3	0	0.3			
	5	3	1	3	1	1	3	0	1	3	0	0.3
	7	3	1	5	1	1	5	0	1	2	0	0.3

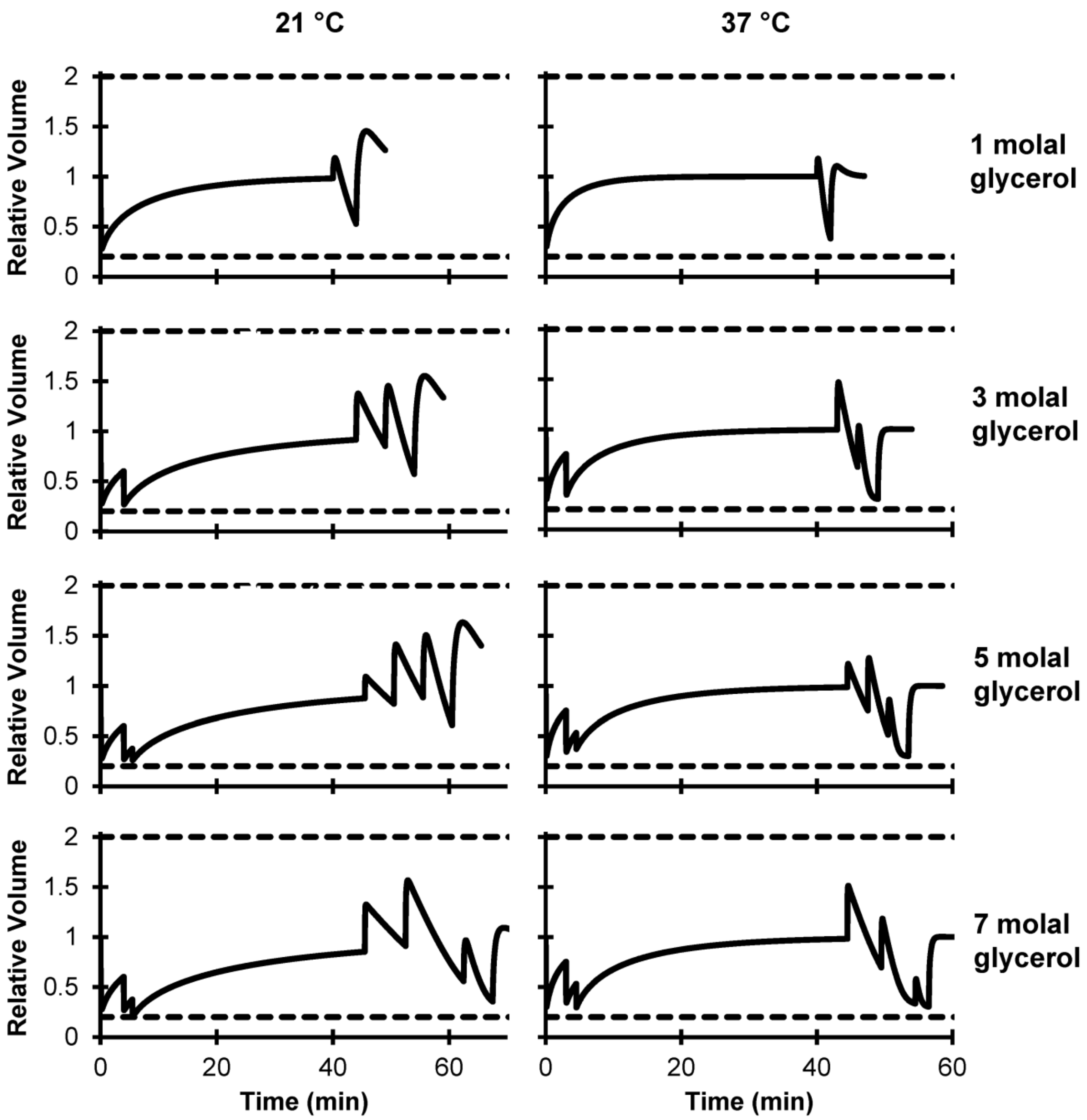

Fig. S1. Predicted cell volume excursions during multistep CPA addition and removal for CPA cytotoxicity studies. The relative osmotically active volume ($\overline{V}_w + \overline{V}_s$) was predicted at the indicated temperature (21 °C or 37 °C) for a 40 min exposure to the indicated peak glycerol concentration (1, 3, 5 or 7 molal). This is the longest exposure time used in the cytotoxicity studies and represents the most extreme changes in the predicted cell volume. In all cases, the predicted cell volumes remain within the osmotic tolerance limits (dashed lines).

Predicted Cell Volume Changes for Single Step and Conventional Multistep Procedures

As a basis for comparison to our mathematically optimized procedures we also carried out single step and conventional multistep procedures for addition and removal of 17 molal glycerol. Table S3 shows the solution compositions and exposure times for these procedures, and Fig. S2 shows the predicted cell volume changes. While cell volumes are predicted to exceed the osmotic

tolerance limits for the single step procedure, the multistep procedure is predicted to maintain the cell volume within the osmotic tolerance limits.

Table S3. Single step and conventional multistep procedures for addition and removal of 17 molal (mol/kg water) glycerol.

Procedure		Step	Glycerol (mol/kg)	Nonpermeating solute (Osm/kg)	Time (min)
Single-Step	Addition	1	17	0.68	15
	Removal	1	0	0.30	30
Conventional Multi-Step	Addition	1	1.5	0.33	6
		2	9.0	0.49	12
		3	17	0.68	10
	Removal	1	4.0	1.0	5
		2	1.5	1.0	5
		3	0	1.0	5
		4	0	0.30	3

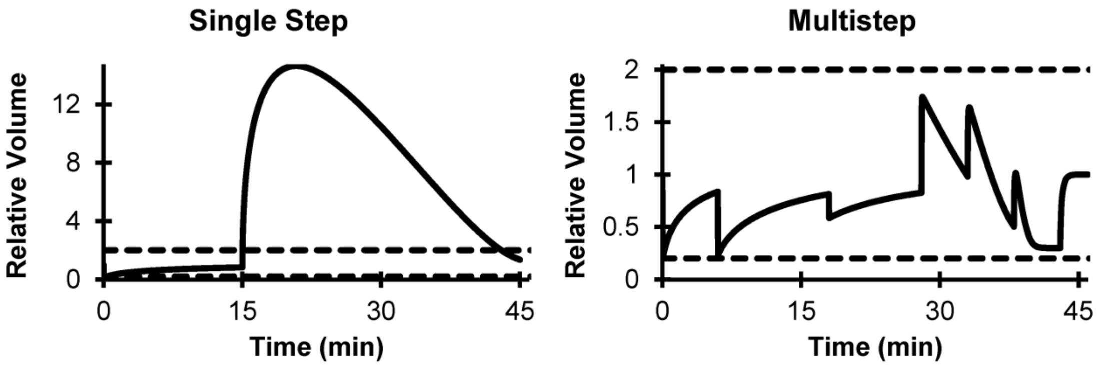

Fig. S2. Relative osmotically active cell volume ($\overline{V}_w + \overline{V}_s$) during single step and conventional multistep procedures for addition and removal of 17 molal glycerol.

References

1. Fry AK, Higgins AZ. Measurement of Cryoprotectant Permeability in Adherent Endothelial Cells and Applications to Cryopreservation. Cell Mol Bioeng. 2012;5(3):287-98. doi: 10.1007/s12195-012-0235-x.
